# Five Questions about Mycoviruses

**DOI:** 10.1371/journal.ppat.1005172

**Published:** 2015-11-05

**Authors:** Moonil Son, Jisuk Yu, Kook-Hyung Kim

**Affiliations:** 1 Department of Agricultural Biotechnology and Center for Fungal Pathogenesis, Seoul National University, Seoul, Korea; 2 Research Institute of Agriculture and Life Sciences, Seoul National University, Seoul, Korea; 3 Plant Genomics and Breeding Institute, Seoul National University, Seoul, Korea; Duke University Medical Center, UNITED STATES

## What Are Mycoviruses?

Mycoviruses are viruses that infect fungi. The first mycovirus was reported in 1962 from the cultivated mushroom, *Agaricus bisporus*; the infected mushrooms developed malformed fruiting bodies, grew slowly, and matured early, resulting in serious yield losses [1]. Like viruses that infect animals and plants, mycoviruses require the living cells of other organisms to replicate. While sharing some characteristics with animal and plant viruses, mycoviruses also have the following unique characteristics: (1) most mycoviruses lack an extracellular route for infection; (2) mycoviruses are transmitted intercellularly only through cell division, sporulation, and cell fusion; and (3) mycoviruses apparently lack a movement protein, which is essential for the life cycle of animal and plant viruses.

According to the most recent report concerning virus taxonomy, the genome of most mycoviruses consists of double-stranded RNA (dsRNA), while the genome of about 30% of mycoviruses is composed of a positive, single-stranded RNA (+ssRNA) [2]. A geminivirus-related DNA mycovirus was recently reported for the first time [3]. Mycoviruses have been detected in all of the major phyla of fungi, including the Chytridiomycota, Zygomycota, Ascomycota, Deuteromycota, and Basidiomycota [1]. Although many mycoviruses and their host fungi have been identified, many mycoviruses undoubtedly remain unknown. Recently developed metagenomic approaches will be useful for detecting and identifying new mycoviruses.

## How Did Mycoviruses Arise?

Mycoviruses have been classified into seven linear dsRNA families *(Chrysoviridae*, *Endornaviridae*, *Megabirnaviridae*, *Quadriviridae*, *Partitiviridae*, *Reoviridae*, and *Totiviridae*), five linear positive-sense ssRNA families (*Alphaflexiviridae*, *Barnaviridae*, *Gammaflexiviridae*, *Hypoviridae*, and *Narnaviridae*), unclassified linear negative-sense ssRNA, and circular ssDNA virus [1]. Mycoviruses have usually been detected by the purification of dsRNA molecules because many mycoviruses produce dsRNA or dsRNA replicative intermediates in their fungal hosts [4]. The profiling of purified dsRNA has demonstrated the diversity of mycoviruses. Several dsRNA-containing fungal isolates showed multiple dsRNA patterns that might represent segmented viral genomes, mixed infections of more than two viruses, or defective RNAs [4,5]. The levels of dsRNA and/or ssRNA mycoviruses may be overestimated, however, by the use of dsRNA-enriching protocols.

Phylogenetic studies have demonstrated that viruses in the same taxonomic families can infect diverse hosts, including fungi, plants, animals, and protozoa [1,6]. For example, a recent taxonomic review indicated that the family *Partitiviridae* contained dsRNA viruses that infect plants, fungi, or protozoa [7]. In addition, the positive-strand RNA mycoviruses, which include Cryphonectria parasitica hypovirus 1–4 (CHV1–4), Fusarium graminearum virus 1 (FgV1), and Botrytis virus X, are phylogenetically related to plant viruses. Their genomic organization and expression strategy resemble those of plant potyviruses or potex-like viruses [1,8]. Moreover, Sclerotinia scerotiorum RNA virus L is closely related to the human pathogen hepatitis E virus and rubi-like viruses [3].

Two major hypotheses have been proposed to explain the origin of mycoviruses [4]. The “ancient coevolution hypothesis” states that although the origin of mycoviruses is unknown, the association between mycoviruses and fungi is ancient and reflects long-term coevolution. The “plant virus hypothesis,” in contrast, suggests that mycoviruses originated relatively recently from plant viruses; i.e., the original mycovirus was a plant virus that moved from plant to fungus within the same host plant. Similar scenarios might also explain the origin of plant viruses; i.e., some plant viruses may have originated from mycoviruses that moved from fungus to plant [7]. Because convincing data are lacking, however, the origin of mycoviruses remains a mystery.

## What Do Mycoviruses Do in Their Fungal Hosts?

Although mycoviruses are common among fungi, they usually remain latent and seldom induce symptoms [4]. Some mycoviruses, however, cause dramatic changes in their hosts, including irregular growth, abnormal pigmentation, and altered sexual reproduction [1,3,4,9]. Perhaps the most important effect is the reduced virulence—i.e., hypovirulence—of plant-pathogenic fungi. Hypovirulence has attracted much attention because it has the potential to reduce the losses of crops and forests caused by plant-pathogenic fungi [9,10].

Over the last 50 years, research on mycoviruses that induce hypovirulence has greatly increased our understanding of mycoviruses and their interactions with their plant-pathogenic fungal hosts [1]. Much of the early research on mycoviruses concerned the interaction between hypovirus CHV1 and the chestnut blight fungus *Cryphonectria parasitica*. Infection by CHV1 resulted in reduced growth and abnormal pigmentation in *C*. *parasitica*. Most importantly, CHV1 induced hypovirulence in *C*. *parasitica* [9,11]. Along with the CHV1, the mycoviruses that infect the important plant-pathogenic fungus *F*. *graminearum* have also been detected and studied [12]. Among them, FgV1 confers hypovirulence to *F*. *graminearum* just as CHV1 confers hypovirulence to *C*. *parasitica* [12]. When infected by FgV1, *F*. *graminearum* shows decreased vegetative growth, abnormal pigmentation, and reduced mycotoxin production [12]. Interestingly, FgV1 can also be transmitted to *C*. *parasitica* and to other *Fusarium* species, and FgV1 induces more severe hypovirulence than CHV1 in *C*. *parasitica* [13].

In addition to the mycoviruses mentioned in previous paragraph, several hypovirulence-associated mycoviruses, such as Sclerotinia sclerotiorum hypoviruses, Helminthosporium victoriae viruses, and Rosellinia necatrix viruses, have been detected and studied using reverse genetic approaches [4,10]. Because these viruses lack an extracellular phase, researchers have investigated transfection methods using purified virus particles, full-length viral cDNA clones, and in vitro RNA transcripts [14–16]. These infection assays will facilitate identification of viral and/or host factor(s) involved in symptom induction or virus replication for many mycovirus–host systems. These methods can also be used to expand the host ranges of some mycoviruses.

As mentioned earlier, mycoviruses are transmitted intercellularly only through hyphal anastomosis or spores. Virus transmission between different strains is restricted by fungal vegetative incompatibility (vic). Vegetative incompatibility is an obstacle in the use of hypovirulent mycoviruses as biological control agents. Recent research has demonstrated that the seven *vic* genes associated with five of six vic loci in *C*. *parasitica* contribute to incompatibility and affect virus transmission [17].

Although much of the research concerning mycoviruses has dealt with hypovirulence of pathogenic fungal hosts, mycoviruses of yeasts and nonpathogenic fungi are also important. Several dsRNA and ssRNA viruses have been found in the yeast *Saccharomyces cerevisiae* [18]. Among them, Saccharomyces cerevisiae virus L-A (ScV-L-A) and its killer toxin-encoding satellites have been well characterized.

## How Do Mycoviruses Change the Expression of Host Genes?

Recently developed analytical techniques have enabled research on mycoviruses to enter a new phase. Using genome-wide linkage analysis, for example, researchers have begun to answer the question, “How do mycoviruses affect their fungal hosts?” RNA-Seq–based, genome-wide expression analysis revealed totally distinct expression patterns of *F*. *graminearum* transcriptomes in response to infections by four phylogenetically different mycoviruses (FgV1–4) [19]. Among these viruses, FgV3 and FgV4 did not cause any visible changes in the phenotypes of the host fungus even though they caused equal or greater changes in transcriptome expression levels than FgV1 and FgV2, which did cause visible changes in the host phenotype [19]. Further detailed study will undoubtedly increase our understanding of the interactions between mycoviruses and their hosts.

As obligate intracellular parasites, mycoviruses reprogram host cell metabolism in order to replicate within host cells and avoid antiviral responses. Identifying the crucial determinants in all steps of the viral life cycle is important for understanding the pathology caused by mycoviruses [20]. To identify host factors important in the interaction between mycovirus and fungus, researchers have used genome-wide approaches in their studies of Cryphonectria hypoviruses, Fusarium graminearum viruses, and Sclerotinia sclerotiorum debilitation-associated RNA virus [19–22]. The results revealed that the expression level of specific host genes differed not only between virus-free and virus-infected fungus isolates but also among viruses belonging to different groups and among virus strains that differed in the degree of hypovirulence that they induced.

These data indicate that mycoviruses depend on various host factors as well as cellular processes and pathways, including those related to metabolism, cellular transport, RNA processing, and signaling. The host genes that have been analyzed for biological function in mycovirus–host interactions are listed in Table 1. Among these host genes in *C*. *parasitica*, the transcription factors *cpst12* and *pro1* are down-regulated by CHV1 infection and are related to female sterility and viral replication, respectively [23]. In *F*. *graminearum*, the host gene *hex1* is up-regulated by FgV1 infection and is associated with the accumulation of FgV1 RNA in host cells [24]. Viral RNA accumulation is decreased in Δ*hex1* and increased in the overexpression mutant compared to the wild type [24]. In yeast, the host protein Mak3p, which is an N-acetyl transferase, is required for acetylation of the major coat protein of ScV-L-A, and such acetylation is necessary for virus assembly. In addition, superkiller (Ski) proteins of yeast have anti-RNA virus activities [18]. Taken together, the combination of genome-wide and single-gene investigation will provide a more comprehensive understanding of the interactions between mycoviruses and fungal hosts.

**Table 1:** Host genes involved in interactions between mycoviruses and host fungi.

Fungus	Fungal host gene	Mycovirus	Function involved in	Ref.
*C*. *parasitica*	*Pro1*	CHV1	stable inheritance of CHV1	[23]
	*Bir*	CHVs	transmission of the hypoviruses	[33]
	*MK1*,*PK1*	CHV1	CHV1 symptom development	[34]
	*NAM-1*	CHV1	CHV1-induced symptom expression	[35]
*H*. *victoriae*	*Hv-p68*	HV145S	accumulation of Hv145S	[36]
*S*. *sclerotiorum*	*SsITL*	SsDRV	suppression of host resistance for SsDRV	[37]
*F*. *graminearum*	*FgHex1*	FgV1	accumulation of FgV1 viral RNA	[24]
	*FgHal2*	FgV1	accumulation of FgV1 viral RNA, transmission of FgV1	[38]
*S*. *cerevisiae*	*Mak3*	ScV-L-A	ScV-L-A assembly	[18]
	*Ski1*	ScV-L-A	degradation of ScV-L-A viral RNA	[18]

RNA silencing, which is termed quelling in fungi [25], has been studied in depth in the model fungus *Neurospora crassa*, and virus-induced quelling has been reported in *C*. *parasitica* and *Aspergillus nidulans* [25]. Because mycovirus genomes consist of RNA, quelling has obvious potential for fungal defense against mycovirus infection. In *C*. *parasitica*, *dcl2* and *agl2* genes, which are required for RNA-silencing antiviral defense responses, are induced by CHV1 infection [26]. Significant changes of expression level of some silencing-related genes (*rdr1*, *dcl1*, *dcl2*, and *agl2*) upon virus infection were also observed in Fusarium gramineraum viruses-infected *F*. *graminearum* [19]. Transcript accumulation levels of *dcl2* and *agl2* were decreased only in FgV1-infected *F*. *graminearum*, whereas accumulation levels of these transcripts were increased by FgV2–4 infections [19]. Therefore, it seems possible that RNA silencing pathways can be induced by mycoviruses. The key RNAi components responsible for the regulation of this antiviral mechanism in *F*. *graminearum* remain unclear. To reduce a host’s virus defense responses, many viruses, including mycoviruses, produce silencing suppressors that incapacitate RNA silencing. For example, the p29 protein of mycovirus CHV1 and the *S10* gene product of Rosellinia necatrix mycoreovirus 3 function as silencing suppressors [11,27].

## Should Mycoviruses Be Regarded As Harmful or Beneficial?

Most of the initial research on mycoviruses concerned their identification and their effects on commercial mushrooms and other valued fungi. Although mycoviruses are considered undesirable when they attack commercial mushrooms, they are considered beneficial when they act as biological control agents of fungal pathogens in economically important plants. Several approaches using hypovirulent strains have been attempted to manage fungal diseases in plants [3,9,10]. In the 1980s, spores of *C*. *parasitica* containing hypovirus were artificially introduced into fungal populations to control chestnut blight. This approach completely failed in forests but was successful in orchards in eastern North America and in Europe [9]. These differences in efficacy resulted from differences in vegetative compatibility among fungal isolates or from the properties of the hypoviruses [17]. These results suggest that development of mycoviruses as effective biological control agents may require consideration of multiple factors, including both host and virus properties. Sclerotinia sclerotiorum hypovirulence-associated DNA virus 1 was recently applied to control rapeseed stem rot caused by *Sclerotinia sclerotiorum*. This hypovirulent strain suppressed the disease, whether it was applied as a suspension of virus-infected hyphal fragments or virus particles [3,10]. Several challenges must be overcome in the use of hypovirulent strains to control plant-pathogenic fungi. As noted previously, one challenge is vegetative incompatibility, which prevents mycovirus transmission from a hypovirulent strain to a target strain. Another challenge is the potential lack of fitness of the hypovirulent strain. These barriers to achieving biological control with mycoviruses are likely to be overcome as research increases our understanding of mycoviruses and their interactions with host fungi and the environment [17].

As mentioned earlier, our understanding of the interactions between mycoviruses and their fungal hosts has been enriched by research on those mycoviruses that induce hypovirulence [9,10]. However, many mycoviruses do not significantly affect their fungal hosts. This suggests that these viruses may be adapted to living with their hosts for long periods and that the association may even benefit both mycovirus and fungus [28,29]. In yeast, “the killer phenomenon” is caused by the combined presence of cytoplasmically inherited dsRNA virus and satellites or DNA virus-like elements (VLEs) [18]. Although these viruses do not induce symptoms in their hosts, they do substantially affect host biology. A recent report suggested that killer systems are so beneficial to their hosts that they have resulted in the loss of host RNAi systems [30]. In another recent report, Kast et al. [31] found that the auto-selection of cytoplasmic virus-like elements encoding toxin/antitoxin systems in the yeasts *Pichia acaciae* and *Kluyveromyces lactis* involves a nuclear barrier for immunity gene expression. These results indicate that symptomless or latent mycoviruses may have unknown functions in their hosts. Researchers have also described a three-way symbiotic relationship among a mycovirus, an endophytic fungus, and tropical panic grass; in the absence of the mycovirus, the endophytic fungus and grass cannot survive high soil temperatures [32]. Other mycoviruses that benefit their hosts probably remain to be discovered.

Although 50 years of research has enriched our understanding of mycoviruses, researchers still do not know how to initiate infection so as to determine cause and effect with respect to mycoviruses. As noted earlier, transfection methods and reverse genetic systems have been developed for several mycoviruses [14–16]. The future development of reverse genetics systems for many other mycoviruses should contribute to our understanding of mycovirus molecular biology and should facilitate the stable application of mycoviruses as biological control agents or as virus-based expression vectors. The reverse genetics system should overcome restrictions to horizontal virus transmission caused by fungal vegetative incompatibility and nonself recognition systems and should thus increase the use of hypoviruses in biological control. In addition, future studies are likely to continue to reveal important clues regarding novel role(s) of mycoviruses in host biology. Supported by continued advances in scientific technology, research on mycoviruses and their fungal hosts will provide new insights into the largely unknown world of mycoviruses.
